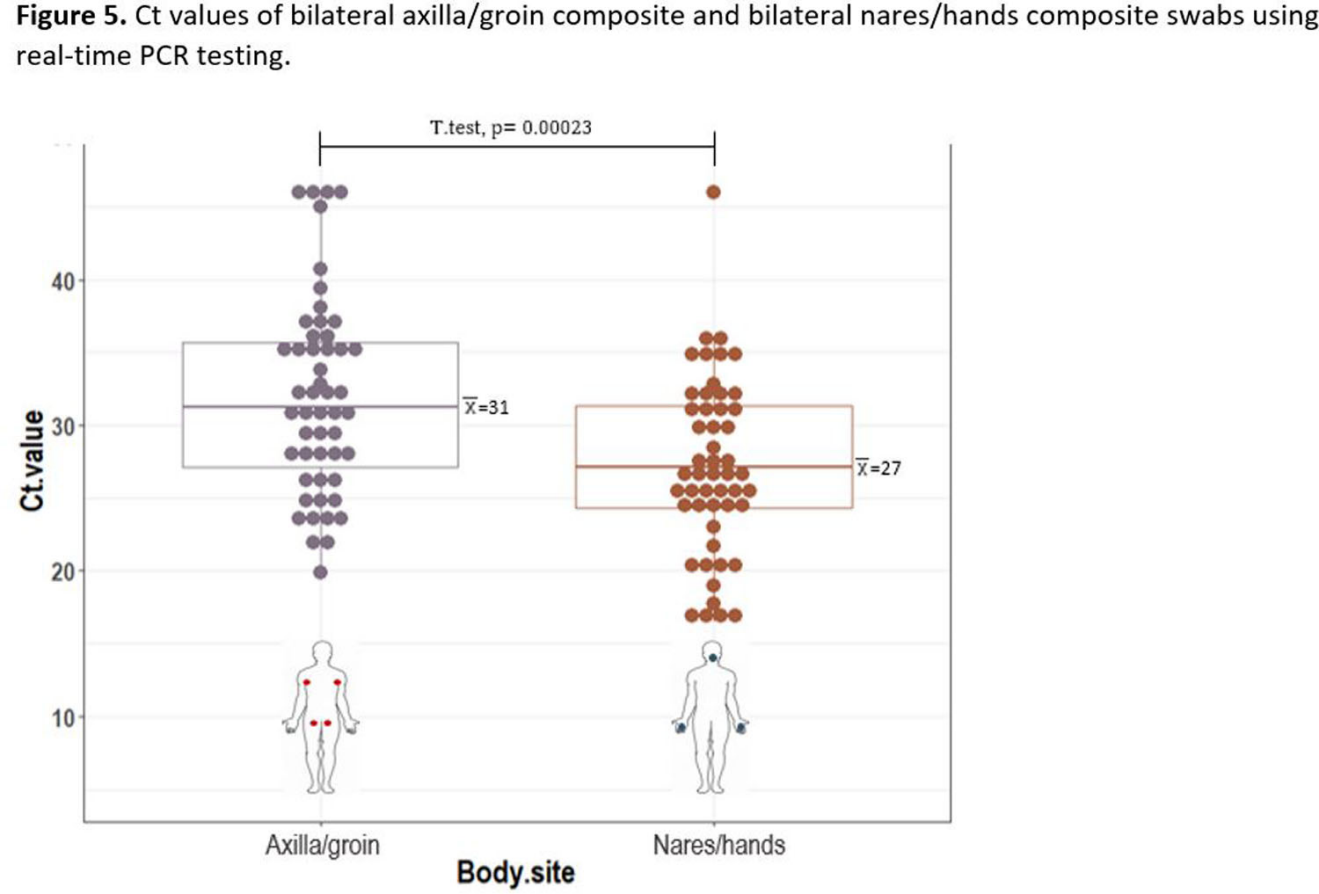# Improving Consistency and Accuracy: A Novel C. auris Colonization Screening Strategy Using a Nares + Hands Composite Swab

**DOI:** 10.1017/ash.2024.268

**Published:** 2024-09-16

**Authors:** Luisa Lopez Cano, Sebastian Arenas, Adriana Jimenez, Meghan Lyman, Anastasia Litvintseva, Bhavarth Shukla, Joe Sexton

**Affiliations:** Centers for Disease Control and Prevention; University of Miami Health System; US Centers for Diseases Control and Prevention

## Abstract

**Background:** Candida auris is often identified in healthcare settings through bilateral composite of axilla/groin skin swabs screening. Re-screening the same patient has demonstrated inconsistent results over time, complicating the understanding of longitudinal colonization and limiting confidence in negative **Results:** Previous studies have described identification of colonized patients using other anatomical sites. Here, we compare bilateral composite of nares/hands with bilateral composite of axilla/groin screenings in a cohort of hospitalized patients in Miami, Florida, to assess the use of screening other body sites for C. auris surveillance. **Methods:** This study took place in a 560-bed academic acute-care facility and included patients previously colonized with C. auris who were cohorted on a 30-bed unit. Bilateral composite samples from both the axilla/groin and nares/hands were obtained simultaneously. Swabs were collected at six different time points at biweekly intervals between March and May 2023 (Figure 1) and sent to the Centers for Disease Control and Prevention for testing with culture and Real-time PCR-based methods. **Results:** A total of 102 swabs (51 from each swab type) were collected from 19 patients who were each sampled a median of twice (IQR: 1-5). Among the 102 swabs, 35 of 51 (69%) axilla/groin swabs were positive compared with 45 of 51 (88%) nares/hands swabs using culture (Figure 2). Furthermore, 48 of 51 (94%) swabs were positive by culture for both methods, with 15 positive from the nares/hands and one positive from the axilla/groin (Figure 3). Among 11 patients who were tested ≥2 times with nares/hands swabs, 9/11 (81%) tested positive on all sequential swabs via culture and 10/11 (90%) tested positive via PCR (Ct threshold < 3 6.9). Among the same 11 patients but using the axilla/groin swabs, 3/11 (27%) patients tested positive on all sequential swabs using culture, and 5/11 (45%) tested positive using PCR (Figures 2-4). On average, samples collected from nares/hands swabs had lower Ct values (mean=27) compared to axilla/groin swabs (mean=31) (p-value=< 0.001) (Figure 5). **Discussion:** Identifying the swab site with most consistent C. auris detection is important for surveillance purposes. In our study, there were more positives and consistent positivity for nares/hands by both culture and PCR-based methods, as well as lower Ct values, suggesting that these swabs provide more reliable detection of C. auris colonization. Alternative screening methods deserve consideration as CDC continues to explore whether swabbing of other body sites (e.g., nares, hands) would improve accuracy and consistency when identifying colonized patients.

**Figure:**
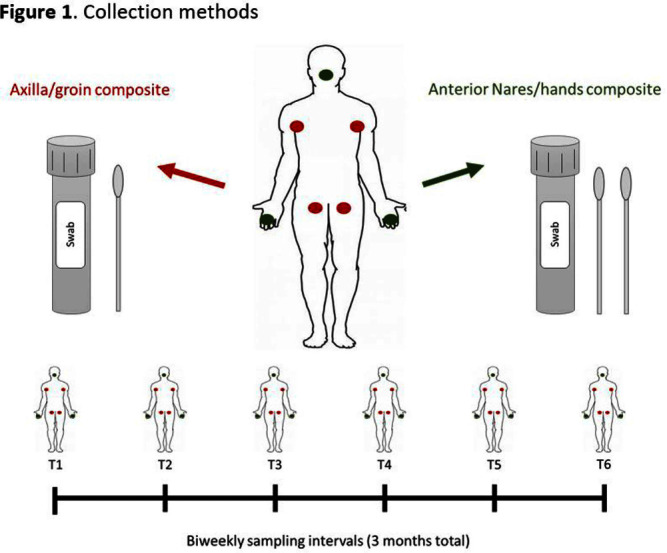

**Figure:**
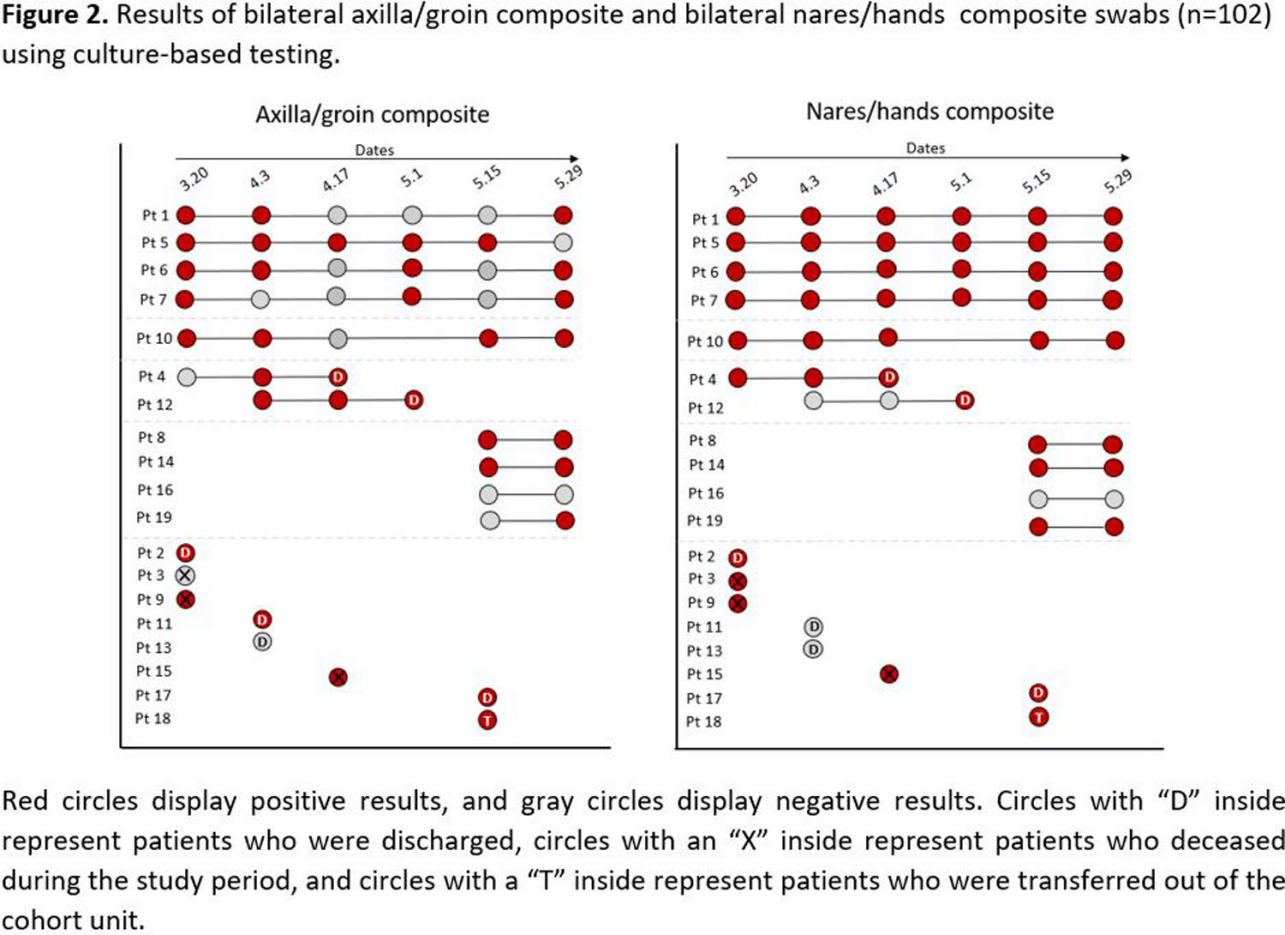

**Figure:**
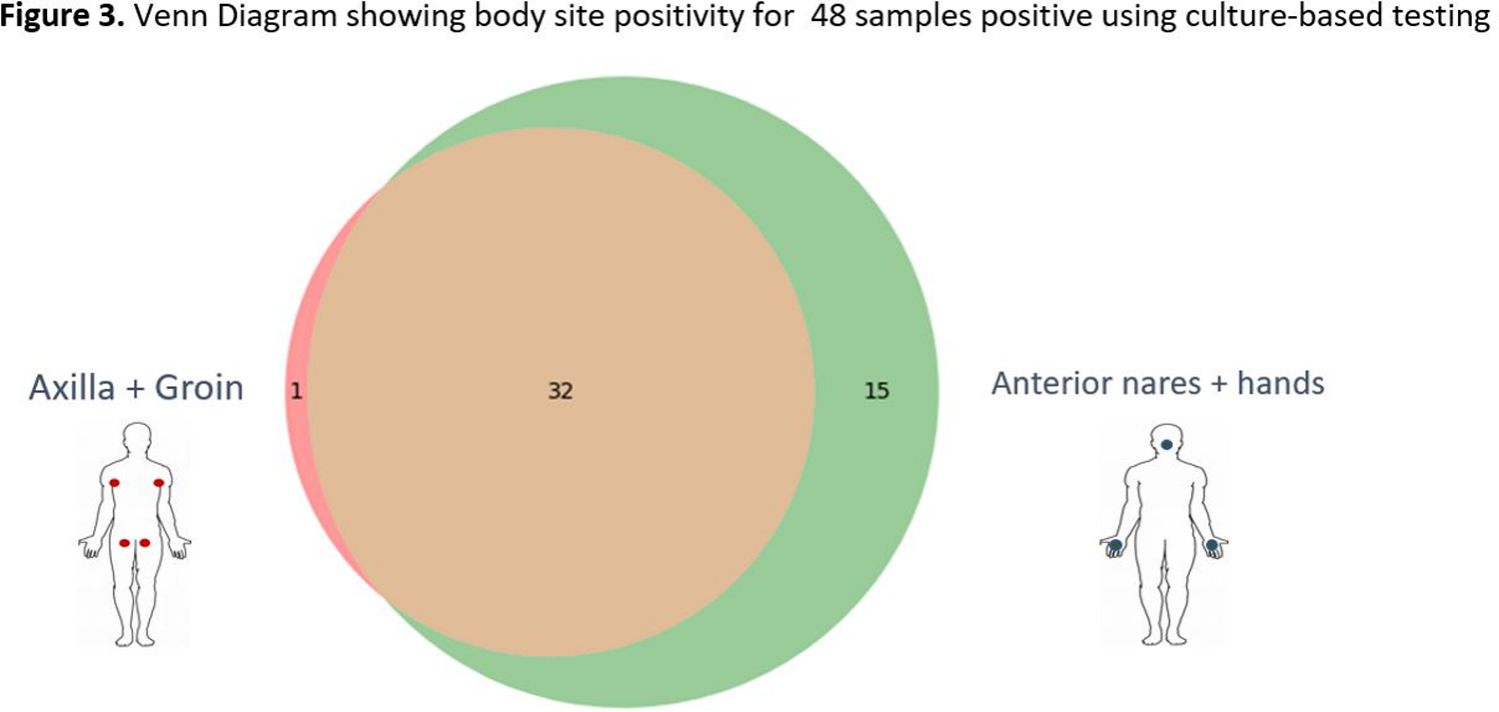

**Figure:**
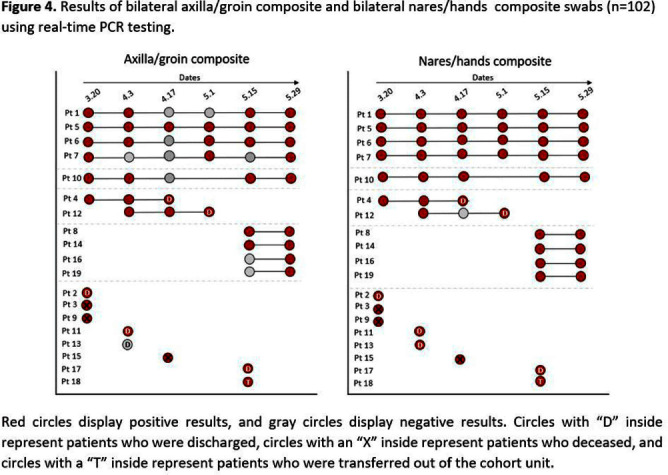

**Figure:**